# Eosinophilic Fasciitis and Smoldering Multiple Myeloma: An Exceptional Association in Young Adults

**DOI:** 10.7759/cureus.23896

**Published:** 2022-04-06

**Authors:** Rajaa Jabbouri, Nouama Bouanani, Rita Aniq Filali, Jehanne Aasfara

**Affiliations:** 1 Department of Internal Medicine, Mohammed VI University of Health Sciences (UM6SS), Casablanca, MAR; 2 Department of Haematology, Mohammed VI University of Health Sciences (UM6SS), Casablanca, MAR; 3 Department of Neurology, Cheikh Khalifa International University Hospital, Faculty of Medicine, Mohammed VI University of Health Sciences (UM6SS), Casablanca, MAR

**Keywords:** hemopathy, corticosteroids, multiple myeloma, eosinophilic fasciitis, shulman's fasciitis

## Abstract

Eosinophilic fasciitis (EF) or Shulman's fasciitis is a rare condition characterized by subcutaneous edematous induration sparing the face and distal extremities and progressing to skin sclerosis. Its association with other pathologies, notably hemopathies, is described in the literature, but its association with smoldering multiple myeloma remains very rare, especially in a younger subject.

## Introduction

Eosinophilic fasciitis (EF) or Shulman's fasciitis is a rare condition first described in 1974 by Lawrence E. Shulman [1]. The diagnosis of the disease is not standardized [2,3] but is based on the presence of the thickening of subcutaneous tissues that may progress to skin sclerosis, sparing the face, hands, and feet, with possible joint involvement without Raynaud's phenomenon. Hypereosinophilia is frequently present but is not mandatory for EF diagnosis [4]. Confirmation is histological with the demonstration of the thickening of the fascia with inflammatory infiltration, mostly composed of lymphocytes and eosinophils. Muscle magnetic resonance imaging (MRI) can also be very suggestive, but it does not replace deep fascial biopsy. Its association with other pathologies has been reported, notably hemopathies in 10% of cases [5,6]. Its association with smoldering multiple myeloma is very rare. Our aim is to highlight the need for a hematological assessment, even in young patients, to look for any hemopathy that may occur concomitantly in the months following the onset of EF or during its remission phase [5].

## Case presentation

**Figure 1:**
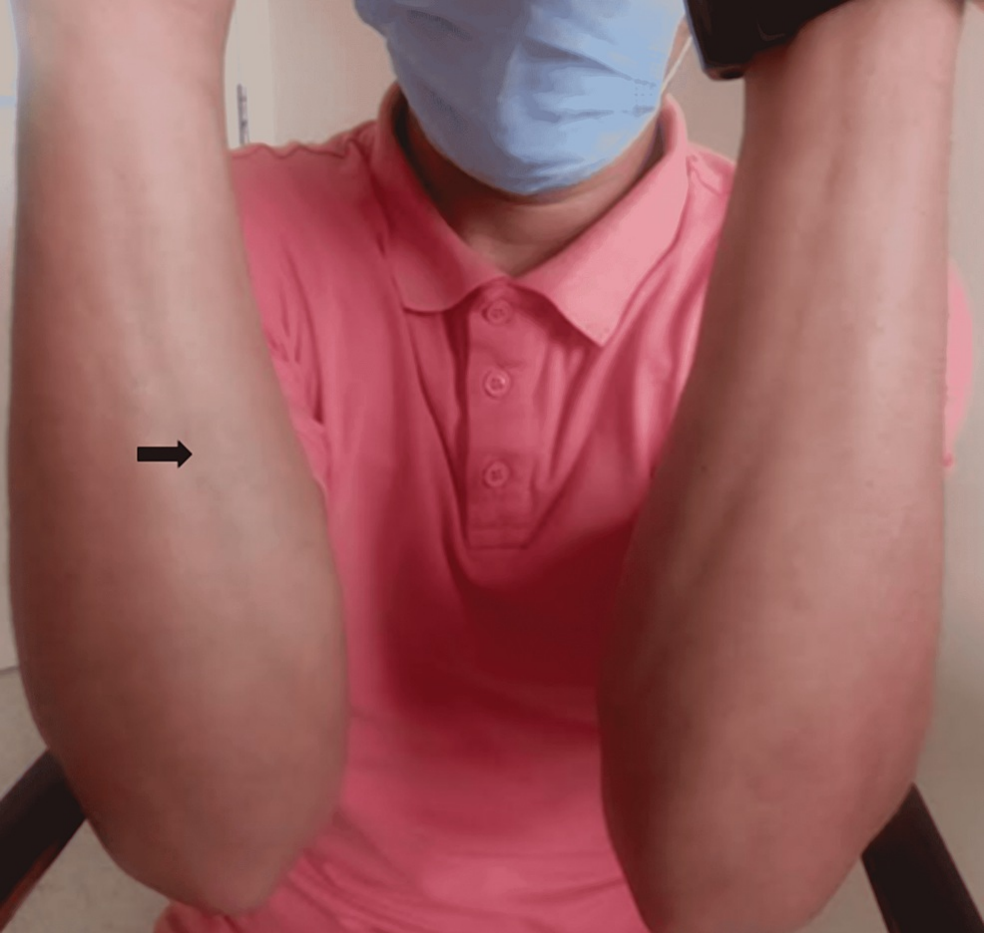
Canyon's sign and orange peel appearance Canyon's sign (also called "sunken vein") - depression along the veins' trajectory (arrow), resulting from the thickening of subcutaneous tissues.

**Figure 2:**
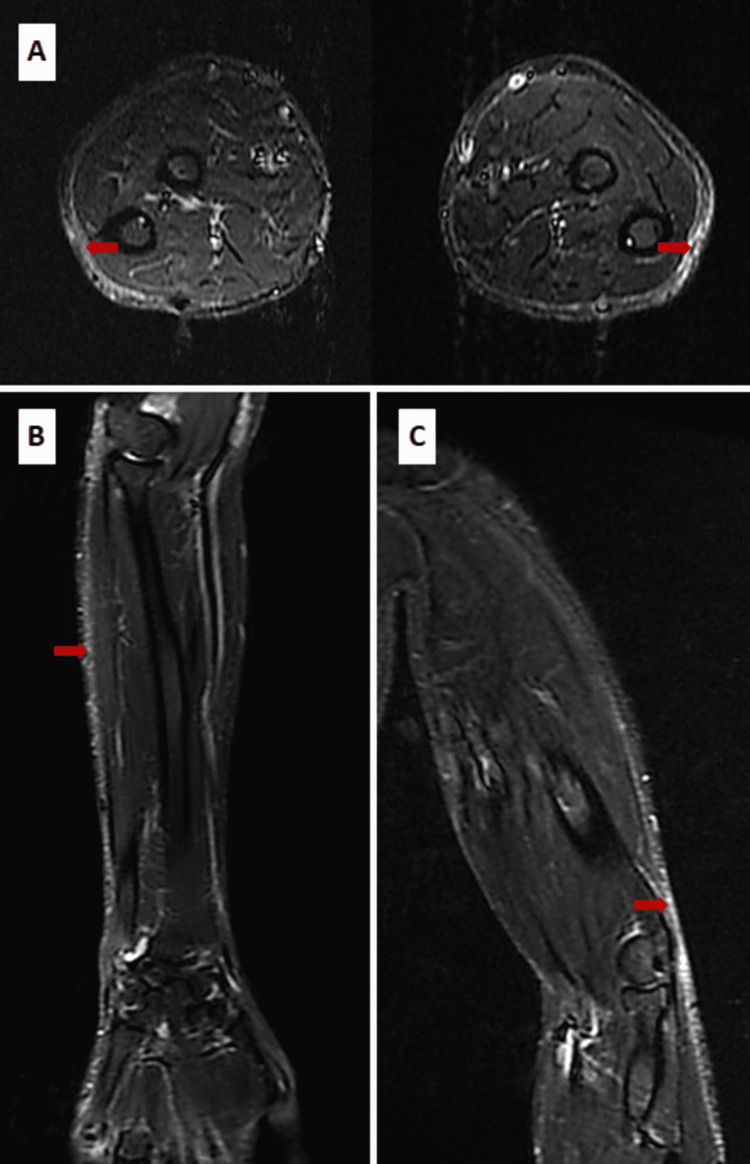
Fasciitis of the upper limbs on MRI Thickening and STIR hypersignal of the deep fasciae (arrows) without muscle damage. Symmetrical involvement of the forearms (A and B) and arms (C).

**Figure 3:**
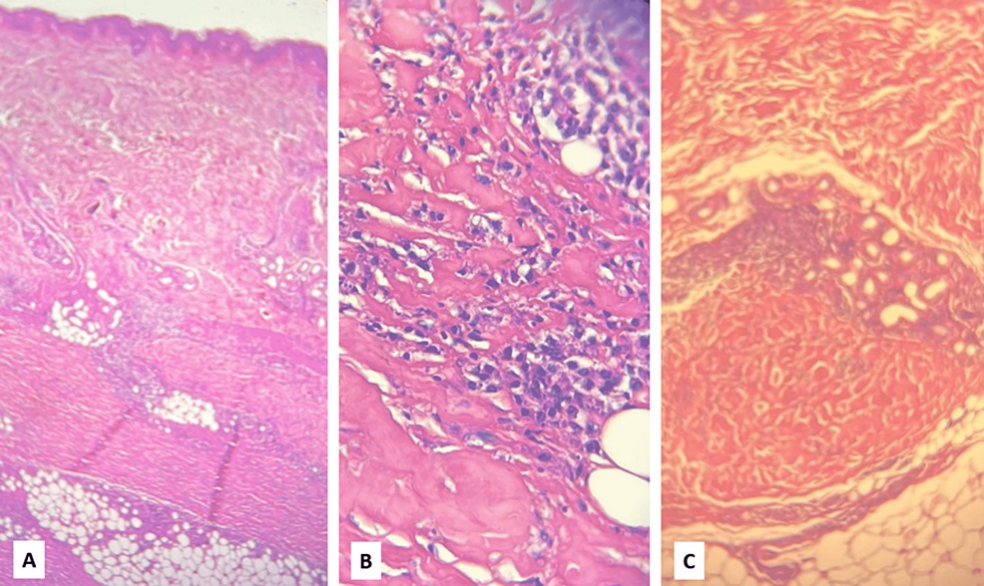
Fibrosing and sclerosing fasciitis: Eosinophilic fasciitis. A: Skin tissue and hypodermis: low magnification view showing an inflammatory infiltrate in the dermis. B and C: High magnification view showing hypodermis and muscle fascia dissociated by collagenous fibrosis with the presence of lymphoplasmacytic inflammatory cells.

A 24-year-old patient, without any particular medical history, presented three months ago with progressive hardening of the skin of the upper limbs, with the onset of a restriction of the joint mobility of the wrists and elbows coupled with inflammatory arthralgias of the metacarpophalangeal joints, wrists, and elbows, without any other associated signs, in particular without any notion of arthritis or Raynaud's phenomenon. He did not relate any subjective dry symptoms either. Clinical examination found the patient in good condition, with a performance status of 0. Dermatological examination revealed induration of the skin on the arms, forearms, and legs, with an "orange peel" aspect on the arms. There was no involvement of the face, hands, or feet. Canyon's sign is present with a linear depression formed by the veins of the arms, which are easily identifiable (Figure 1). Furthermore, there is no evidence of anemia syndrome or bone pain on palpation. Laboratory testing revealed anemia at 11.7 g/dL and hypereosinophilia at 1200/mm^3^ (normal range: 20-630/mm^3^). There was a mild inflammatory syndrome with C-reactive protein at 11.49 mg/L, without increasing serum viscosity (2 mm/hour). The immunological assessment was negative (antinuclear antibody and soluble antibody-antigen complex). MRI of the upper limbs showed thickening of the fascia without inflammatory muscle involvement (Figure 2). A deep skin biopsy was done on the arm, showing dense fibrous thickening of the muscle fascia with an inflammatory infiltrate of lymphocytes and a few eosinophils, confirming the diagnosis of EF (Figure 3). Serum protein electrophoresis showed a monoclonal spike at 66.1 g/dL in the gamma zone. We, therefore, completed it with a serum immunofixation study, which revealed an IgG kappa monoclonal protein. Serum-free light chains were produced in excess with a kappa/lambda ratio of 3.04. The 24-hour proteinuria was negative, and the complete metabolic panel, serum creatinine, and blood calcium were normal. Bone marrow aspiration showed mature and dystrophic plasma cells at 11%. The karyotype was normal, and the fluorescence in situ hybridization (FISH) did not find any mutation, specifically no 17p13 deletion and t(4;14) or t(4;16) translocations. There was no bone involvement as the whole-body MRI was normal. The evaluation was consequently in favor of a smoldering multiple myeloma according to the revised criteria of the International Myeloma Working Group (IMWG). The Revised International Staging System (R-ISS) was evaluated at stage I, and there were no CRAB criteria (anemia, hypercalcemia, renal insufficiency, and bone lesions) to justify the initiation of the treatment for the monoclonal gammopathy. The patient was, however, treated with corticosteroids (prednisone) at a dose of 0.5 mg/kg/day for one month with a decreasing regimen, combined with methotrexate at a dose of 15 mg/week. After two months of treatment, the evolution is marked by a partial regression of the skin sclerosis with recovery of mobility of the wrists and elbows. Regular monitoring of the gammopathy is recommended without any treatment at present.

## Discussion

Eosinophilic fasciitis (EF) or Shulman's fasciitis is a rare condition described by Lawrence E. Shulman in 1974 [1] characterized by a symmetrical edematous induration of the subcutaneous tissues, sparing the face and distal extremities, which can progress to skin fibrosis in about 74% to 96% of cases, giving an orange peel appearance and then a "sunken vein" appearance, also known as the "canyon sign," in about 53% to 80% of patients [7]. The diagnosis of the disease is not standardized [2,3]. However, histological confirmation by deep surgical biopsy, from the skin to the muscle, is necessary even in the presence of suggestive muscle involvement on MRI. Anatomical examination reveals thickening of the fascia with the presence of inflammatory infiltrates made up mainly of lymphocytes and eosinophils (in 50% of cases) [8]. Peripheral hypereosinophilia is frequently found in the acute phase [4] in 63% to 86% of cases, but it is not essential to the diagnosis [9].

EF may be associated with other diseases, mainly hemopathies in about 10% of cases [4-7]. These are mainly global bone marrow aplasia, aplastic anemia, autoimmune thrombocytopenic purpura, myelomonocytic leukemia, Hodgkin's disease, and monoclonal gammopathies. The association of EF and multiple myeloma remains rare in the literature [4,10], unlike monoclonal gammopathies, which are frequently described during EF. Our young patient presents smoldering multiple myeloma, which was discovered fortuitously during the systematic workup requested at the time of the diagnosis of EF and does not require specific treatment for the moment. The link between the two pathologies seems to be unclear [9]. The risk of myeloma in association with EF is not specified in the literature. Therefore, regular clinical and biological monitoring is necessary. There is no consensus on the treatment of EF. Corticosteroids remain the first-line treatment with an initial dose of 0.5 to 1 mg/kg/day of variable duration according to the therapeutic response [11-13]. Immunosuppressive drugs, notably methotrexate, mycophenolate mofetil, cyclosporine, azathioprine, and cyclophosphamide [9,11,12,14,15], are indicated in cortico-resistant cases, in about 30% of cases [16].

Our patient received oral corticosteroid (prednisone) at a dose of 40 mg/day (0.5 mg/kg) with progressive decrease. Oral methotrexate was introduced one month later, during the decrease in corticosteroids, due to the partial clinical and biological improvement (normalization of the inflammatory status).

The association of EF with smoldering myeloma in our patient is not usual given his young age (27 years). Elderly patients have a higher risk of developing a hematological disease associated with EF with a risk estimated at 10% [5,17,18].

Hematological involvement in EF worsens the prognosis of the disease [19,20]. The diagnosis of EF, therefore, implies looking for an associated hemopathy, in particular a monoclonal gammopathy, by a systematic hematological evaluation. This is because hematological complications can occur concomitantly in the months following the onset of EF or during the remission phase [5].

## Conclusions

We report a case of Eosinophilic fasciitis associated with smoldering multiple myeloma discovered fortuitously thanks to the additional hematological assessment requested after the diagnosis of EF and justified by the risk of hematological involvement estimated at 10%. This association remains very rare, especially in young subjects. Early diagnosis is necessary as the prognosis is more severe in the presence of hemopathy.
